# Dyslexia Data Consortium: A Comprehensive Platform for Neuroimaging Data Sharing, Analysis, and Advanced Research in Dyslexia

**DOI:** 10.1007/s12021-025-09747-0

**Published:** 2025-10-08

**Authors:** Rishikesh V. Phatangare, Mark A. Eckert, Li Luo, Kenneth I. Vaden, James Z. Wang, Dyslexia Data Consortium

**Affiliations:** 1 School of Computing, Clemson University, Clemson, USA; 2 Department of Otolaryngology - Head and Neck Surgery, Columbia University, New York, USA; 3 Department of Otolaryngology - Head and Neck Surgery, Medical University of South Carolina, Charleston, USA

**Keywords:** Dyslexia Data Consortium, Neuroimaging Datasets, Data Privacy, Data Quality

## Abstract

Neuroimaging studies have and continue to advance our understanding of the neurobiology of dyslexia. Integration of data from these studies has the potential to replicate findings, deepen understanding through theoretically focused research, and provide for unexpected discovery. This data integration can be important for questions where a sufficiently large and well-defined group of participants is necessary for sufficient experimental power, particularly for a complex disorder where age, language background, and cognitive profiles can impact imaging results. We have developed a data-sharing platform to provide a data repository, image processing resources, and data analysis tools, with an emphasis on data harmonization across retrospective datasets (https://dyslexiadata.org). Here, we summarize data sharing, download, imaging metrics, and quality and privacy considerations in the design of and resources available through this repository. By providing access to a relatively large multisite dataset, researchers can test hypotheses about reading development and disability, test novel data analysis methods, even within the platform, and advance understanding of dyslexia.

**Supplementary Information:**

The online version contains supplementary material available at https://doi.org/10.1007/s12021-025-09747-0.

## Introduction

Developmental dyslexia affects an estimated 5% to 17% of the U.S. population (Cooper et al., 2007; Engbers, 2009). There have been significant advances in our neurobiological understanding of reading development and disability (Gabel et al., 2010; Grigorenko, 2001; Pugh et al., 2001). However, these neuroimaging studies typically involve relatively small datasets. Moreover, these data are often siloed within investigator institutions or different data-sharing repositories, complicating the evaluation and replication of findings (Sethi et al., 2013). Data-sharing platforms are a solution for advancing scientific rigor, replication, and discovery (Bly et al., 2001; Book et al., 2016; Bunevicius, 2015).

General prospective multisite repositories, such as the Adolescent Brain Cognitive Development (ABCD) Study (Karcher & Barch, 2020), can provide data for a broad range of constructs that have been collected across a large number of participants. Similarly, the UK Biobank provides a large dataset for a wide range of constructs in adults. However, there is limited characterization of reading and language abilities in these and other open-access data repositories. General retrospective repositories, such as OSF (Foster & Deardorff, 2017) and Zenodo (Druskat et al., 2021), are widely used open-access repositories that are designed to facilitate data storage, data sharing, and distribution of research results. However, there is no centralized, consistently curated resource that would facilitate the integration and analysis of data across reading-related studies. In addition, the types and state of the data in these general repositories are quite variable. This limits replication and the need for computational resources to process the data. There are well-utilized area-specific prospective and retrospective data repositories, such as the Alzheimer’s Disease Neuroimaging Initiative (ADNI) (Petersen et al., 2010) and the Autism Brain Imaging Data Exchange (ABIDE)(di Martino et al., 2013). These repositories have provided a model for how data can be successfully shared in an open-access context where potential challenges of data privacy and acknowledgment of data contributors have been addressed.

The Dyslexia Data Consortium (DDC) repository addresses a critical need by providing a specialized platform for sharing data from neuroimaging studies on reading development and disability. The repository is designed to be inclusive of a wide range of demographic and behavioral profiles, with an emphasis on data related to reading development and disability. This approach has required an emphasis on data harmonization to facilitate integration and analysis of behavioral, clinical, and demographic measures across datasets (for details, see the Data Standardization section). Users of the repository can examine questions involving operationally defined dyslexia definitions or use a dimensional framework for studying variation in reading abilities and neuroimaging data. The ability to cross-reference data from different studies may reveal neural and behavioral profiles that advance the understanding of dyslexia. Additionally, funding agencies increasingly expect data-sharing plans to maximize public benefit from research investments (Rahaman, 2023). The DDC platform aligns with this policy by providing a structured and secure way to share data among dyslexia researchers. This repository provides a robust, flexible, and scalable foundation for evolving research needs, utilizing the Django framework (Foundation, 2025) for its development.

### Platform Overview

Our previous work provided a general overview and vision for this repository (Bhandari et al., 2023). Here, we provide a detailed account of the technical infrastructure, outlining recent enhancements that optimize data accessibility, analytical capabilities, and user experience. By presenting a comprehensive overview of the platform’s architecture, this work aims to strengthen the foundation for collaborative research, enabling more robust and replicable investigations into the neurobiology of dyslexia.

The platform’s web-based access makes it widely available to researchers and trainees. MRI file uploading, downloading, and data quality checks are done through a simple interface, assuring that even those without extensive technical expertise can contribute to the repository. The integration of different computing resources on the backend, including MATLAB (Inc., 2022), JupyterHub (Kluyver et al., 2016), PyTorch (Paszke et al., 2019), and Clemson University’s Palmetto HPC (High-Performance Computing Cluster), equips users with powerful analytical tools, eliminating their need to set up complex computational environments locally. It provides research opportunities for a broader audience, including early-career researchers or those from institutions that may not have access to the same resources. The platform also integrates software for image processing, such as image segmentation, that streamlines research workflows by enabling direct execution of these tasks within the platform. We have also implementeddeep learning models to identify potential loss of voxels representing brain tissue that can occur with skull-stripping. Clemson University’s Palmetto HPC provides the computational infrastructure required to process large and complex datasets such as neuroimaging data. Its distributed processing capabilities accelerate high-throughput studies, making large-scale analyses feasible.

**Fig. 1:**
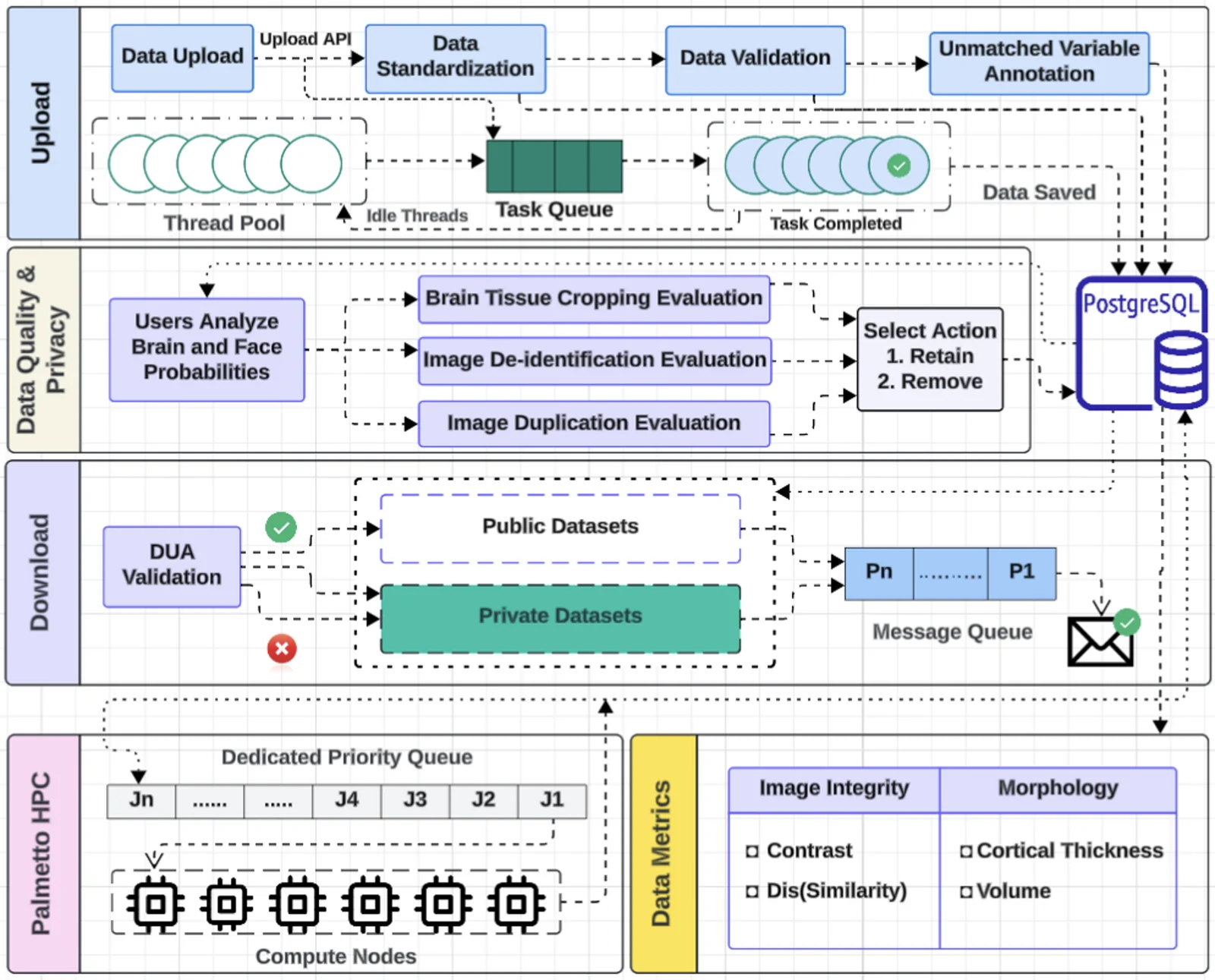
Dyslexia data consortium system architecture. Thread Pool: A group of pre-created processes that run tasks efficiently; PostgreSQL: Database System, HPC: High Performance Cluster, Task Queue: A lineup of tasks waiting to be executed, DUA: Data User Agreement, Compute Nodes: Individual machines that perform processing tasks in an HPC Cluster, Contrast: CAT12 Image Quality Rating, Dis(similarity): Paired Image Correlation approach to identify duplicate images in the Database and Normalization quality, Cortical Thickness: Average Cortical Thickness in HCP MMP1 regions of interest, Volume: Total Gray and White Matter Volumes and gray matter volumes in brain regions linked to dyslexia

The platform’s system architecture (Fig. 1) supports four main functionalities: Data Sharing, Data Download, Data Metrics, and Data Quality & Privacy. The following sections present details of these functions. The subsequent sections examine each of these components in detail, including the strategies adopted to support large-scale neuroimaging analyses on the Palmetto Cluster. Further sections describe the Open-Source Framework for Advanced Neuroimaging Analysis and outline the Data Access Policies and Governance Framework. The manuscript concludes with a summary of the platform’s features and an outline of prospective research directions.

## Data Sharing

Sharing neuroimaging data and related behavioral/demographic files can be challenging for multiple reasons, including the need to link data from participants across different files with data in a repository that might have different variable names. We designed the platform’s data-sharing functionality as a multi-service pipeline that serves users through a pipeline of services. The interface enables users to validate the file formats and metadata and ensure the uploaded data complies with institutional approvals. The DDC is built on the foundational principle of adhering to the Brain Imaging Data Structure (BIDS) standard (Gorgolewski et al., 2016). BIDS offers a standardized directory structure and file-naming convention for organizing neuroimaging and related behavioral data, enhancing human readability, enabling automated processing with modern neuroimaging tools, and supporting inter-study harmonization. All datasets contributed to the Consortium are maintained in BIDS format—either submitted as BIDS-compliant or converted through our platform’s user interface and backend processing scripts.

The data-sharing component (first from the top) in Fig. 1 depicts the workflow for the data-sharing functionality. Each stage of the upload process interacts with the PostgreSQL Database (Douglas & Douglas, 2003) to store user inputs, validation results, mappings, and annotations. This service-to-service redirection model minimizes user effort, enforces data standards, and ensures a streamlined experience from upload to data sharing.

The data-sharing process for the DDC platform consists of the following four steps:

- Data Upload.
- Data Standardization.
- Data Validation.
- Unmatched Variable annotation.

### Data Upload

The interface for the data upload page allows users to upload image data, behavioral data, and demographic data in various file formats. Researchers can upload several images or datasets together via zip/tar files, which is crucial for studies involving large amounts of data or multiple modalities. The interface handles the complexity of uploading and processing large neuroimaging datasets, whether single 3D images (e.g., structural MRI) or 4D image files (e.g., diffusion and functional MRI). It also ensures that the system runs the appropriate back-end scripts for different image types, streamlining the processing of structural MRI (sMRI) images. A user-friendly interface guides researchers through the process, making it easier for non-technical users to interact with the platform. This process minimizes errors in manual data handling, reducing the likelihood of uploading incorrect or unusable data. Missing or mismatched data types are flagged, and related missingness comments are appended to the uploaded behavior file, which is then made available for the user to review. The interface reduces the chances of users uploading errant or incomplete data by offering prompts and checks at each step (e.g., correct file format for neuroimaging, behavioral, and demographic data). Due to the relatively large size of neuroimaging data, the subsequent steps of unzipping the files and saving their content into the database during upload can be time-consuming. To address this, we employ a thread pool that processes these tasks in the background using multithreading. Upon data upload, threads are allocated from the pool to handle file processing asynchronously. The multithreading approach is depicted in the bottom portion of the upload component in Fig. 1. By parallelizing this process, the system avoids blocking the main thread, ensuring that other operations can continue when files are processed. By delaying time-intensive tasks to background threads, users can continue their workflow seamlessly while the platform manages the unzipping and saving in the background. After uploading data, users proceed to the Data Standardization stage to map the uploaded variables to pre-defined database variables. The platform handles the unzipping of large neuroimaging files in the background.

### Data Standardization

Neuroimaging datasets often originate from diverse sources, each employing unique naming conventions and data structures. This variability poses a significant challenge to ensuring consistency and accuracy when integrating and analyzing data across studies. Mismatched or misnamed variables can lead to inadequate statistical modeling, invalid or noisy results, and unreliable conclusions that compromise the integrity of research outcomes. Manually matching variables in large behavioral files, which often include hundreds of entries, is time-intensive and prone to human error. Additionally, the inability to incorporate new or domain-specific variables risks limiting the database’s relevance and adaptability as research evolves. Addressing these challenges requires efficient, automated solutions to standardize behavioral data, align it with existing databases, and uphold data integrity for scalable analyses and reliable results. The Data Standardization service addresses these challenges by presenting uploaded behavioral or demographic variables to the contributor with an intuitive mapping interface. In addition to the interface, the service leverages the Rabin-Karp string-matching algorithm to suggest mappings to predefined database variables. We refer to this process as data harmonization, as it aligns diverse behavioral, clinical, and demographic measures to a common standard.

This *harmonization* process significantly reduces the time and effort researchers would otherwise spend on manual variable matching, enabling them to focus more on their analyses and less on tedious data preprocessing tasks. Variable matching ensures data adheres to consistent definitions and naming conventions, making data integration and comparison across different studies easier. In cases where variables do not exist in the database, users may propose new variable names. These proposed variables are automatically flagged and sent to a DDC administrator for review to determine whether to integrate them into the database. This feature is crucial for maintaining the database’s relevance and flexibility as research methods and neuroimaging modalities evolve. By allowing users to suggest new variables, the platform ensures the possibility of accommodating novel, domain-specific data. The review process also ensures that only well-defined variables are added, thereby preserving the database’s integrity and preventing data redundancy or inconsistency. Once all variables are mapped, users advance to a Data Validation Step.

### Data Validation

Managing and validating research datasets presents several challenges, including ensuring accurate variable mapping between variable names provided by contributors and those in the database, detecting missing or incorrect values, and assuring contributors that their data have been accurately integrated into a repository. Users may inadvertently leave cells empty or input erroneous values, potentially leading to inconsistencies or errors in downstream analyses. Traditional correction methods, such as downloading, editing, and re-uploading files, are time-consuming and prone to mistakes. These issues can undermine the integrity of the data, making it less reliable for research purposes. The data validation service addresses this problem by generating a spreadsheet listing the uploaded variables alongside their mappings.

Users review this information to ensure the mappings are correctly saved. If discrepancies are found, users can revise their mappings. The web-based user interface highlights empty or erroneous cells in red to help users quickly identify and correct these issues. The platform integrates an in-browser spreadsheet editor with auto-saving functionality, so users do not need to download, modify, and re-upload files to facilitate contributor stewardship of their data. Additionally, while some cells may remain empty due to unavailable data for specific participants, users are prompted to confirm these omissions to ensure they are intentional or expected. Once mappings are finalized, users proceed to the Variable Annotation Service. The interface for the Data Validation process is shown in Figure S5 of Online Resource 1.

### Unmatched Variable Annotation Service

The Unmatched Variable Annotation Service identifies variables in a contributor’s dataset that do not match variables in the repository during the data standardization stage and displays them in a spreadsheet. At this stage, the web-based user interface prompts users to provide detailed information for variables that did not automatically match during the standardization process. This step ensures that all non-standard variable names are clearly defined and added to the database. These descriptions undergo manual verification by an administrator to maintain consistency and accuracy across the dataset. By verifying these variables, the platform ensures that all data aligns with the predefined standards, facilitating consistent analysis across different studies. An interface for this feature is shown in Figure S1 of Online Resource 1.

### Data Sharing Preferences

The final step in the data upload process is for users to specify if and how their data can be shared with other users. Contributors are encouraged to share their data openly but can request limited access. 

This flexibility fosters collaboration, where researchers can share their data with specific groups while complying with their institutional policies, where focused data sharing may be allowed in specific situations but not in an open-access context.

## Image Processing and Data Metrics

The platform automatically calculates image data metrics to help users assess data quality and support their research. These data metrics have been used in prior studies to help researchers replicate findings and assess the generalizability of previous results across diverse populations. Here, the goal is to enhance the reproducibility of dyslexia research, support hypothesis-driven exploration, provide an incentive for data sharing, and ultimately advance the scientific understanding of dyslexia.

### Role of Neuroimaging Metrics

The selection of specific data metrics is guided by evidence from neuroimaging studies demonstrating consistent structural and functional alterations in individuals with dyslexia. For example, neuroimaging studies have revealed structural differences in the superior temporal sulcus (STS) and the orbitofrontal cortex (OFC); lower gray matter volume was observed in people with reading disability compared to control participants based on a meta-analysis of the extant literature and direct data analysis of multi-site data in the repository(Eckert et al., 2016b) Regions of interest (ROI) for these STS and OFC are used to collect gray matter volume data from each uploaded image so that users can access these variables for their research. Variables are added to the image processing pipeline and database as reported in the literature and requested by users.

### Dashboard for Neuroimaging Metrics

The neuroimaging metrics are accessed through our platform with a dashboard tool that displays metrics for all datasets stored on the server. This feature allows users to visualize variable values from their dataset(s) with the overall data distribution of the platform. The dashboard generates histograms for each metric, displaying the minimum, maximum, user-observed value, and the mean of the datasets. The dashboard also aids in the identification of trends, anomalies, and data patterns, fostering more informed use of the data. Researchers can better assess how their observations align with or differ from the broader dataset, enhancing the rigor of their analysis.

### Scaling Neuroimaging Analysis with HPC

Significant computational resources are often needed for processing neuroimaging datasets. The DDC platform leverages Clemson University’s Palmetto HPC (High-Performance Computing Cluster), enabling researchers to efficiently process large volumes of data, which would be time-consuming or unable to handle on standard computing systems. This cluster computing infrastructure allows researchers to scale their work by processing multiple datasets in parallel to increase productivity. The Palmetto HPC also reduces processing time, generating results faster, a crucial factor in fast-paced research environments. Our platform employs a standardized data processing pipeline on the Palmetto HPC using established neuroimaging tools like SPM25 (Tierney et al., 2025) and CAT12. Anatomical processing is performed using the CAT12 toolbox within SPM12. All T1-weighted images are spatially normalized to the MNI152NLin2009cAsym template space using geodesic shooting registration. Cortical thickness measures are collected for regions of interest from the Human Connectome Project MMP1 atlas (Glasser et al., 2016). In addition, we collect gray matter volume data from brain regions that have been consistently associated with reading disability to facilitate replication (Eckert et al., 2016b). By maintaining a consistent processing pipeline, researchers can more easily reproduce results across different studies, strengthening the reliability of their findings.

#### High-Performance Computing Workflow 

**Fig. 2:**
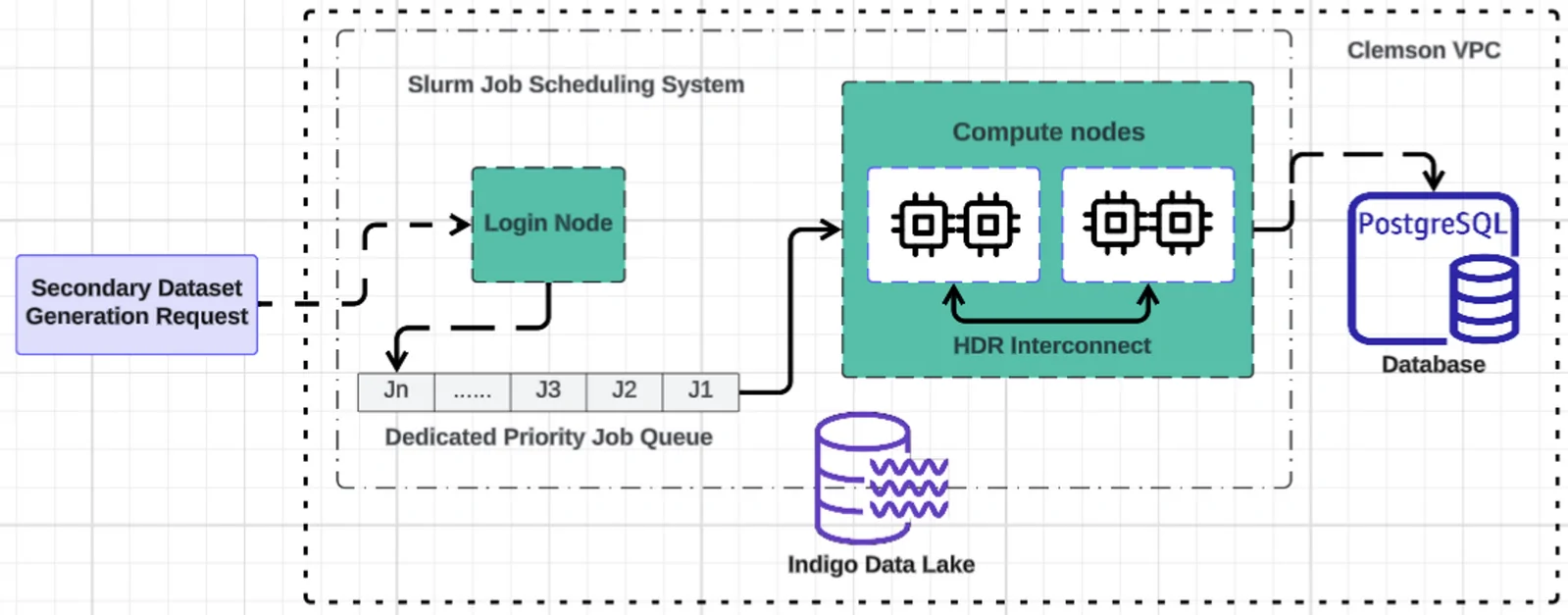
Secondary dataset generation process on Palmetto HPC. The SLURM scheduling system manages job submissions across login nodes and compute nodes, with the Indigo Data Lake storing large datasets, the HDR interconnect enables high-speed data transfer

The Palmetto HPC utilizes the SLURM (Simple Linux Utility for Resource Management – A Cluster Resource Management System) job scheduling system to manage and optimize computational tasks. A dedicated queue is allocated for our jobs, ensuring that computational needs are efficiently met. When a job is submitted, SLURM assesses available resources and dispatches the next job in the queue based on priority and resource availability. Jobs are created on the DDC platform , forwarded to the dedicated queue on the HPC, and executed on a first-come, first-served basis by computing nodes. Figure 2 illustrates the workflow for managing and processing uploaded datasets through the SLURM system. This structured job scheduling process ensures the efficient handling of large datasets and supports scalable research efforts. Effectively queuing and prioritizing jobs allows the system to optimally utilize computing resources, minimizing idle time and maximizing HPC throughput. Real-time updates are made to the database throughout the data pipeline, making sure that the data remains current and accurate for ongoing analysis. In addition, the image processing software has been containerized, providing a stable environment for processing neuroimaging data despite software updates and regular maintenance of the Palmetto HPC. The Palmetto HPC supports hundreds of concurrent jobs across multiple partitions. Jobs are submitted to a dedicated partition that typically allows up to 30 concurrent jobs per user. Average queue times range from 5 to 30 min under normal load but may vary depending on cluster usage. Our Palmetto HPC partition is presently configured for 2-hour wall times, it can be readily modified to support longer runtimes when needed. In addition, additional computational resources such as higher RAM and more CPUs can be allocated to this partition if required. This flexibility ensures that more computationally intensive tools can be accommodated within the same partition as the platform evolves.

The Palmetto Cluster processes data using MATLAB scripts across multiple nodes, enabling the parallel generation of secondary datasets. Running MATLAB scripts in parallel across many nodes accelerates image processing. Distributing these tasks across multiple nodes balances the workload, reduces processing times, and increases efficiency. After processing, the secondary datasets are compressed into a single file and transferred back to the server hosting the platform. Once the data is available on the server, it is ready for use by researchers, who can then download, analyze, and integrate it into their studies. By leveraging the Palmetto Cluster, the DDC platform enables researchers to access advanced computational resources and benefit from a standardized and replicable data processing pipeline, with future enhancements that will allow for even more precise and tailored data analysis. Development and validation of these pipelines will proceed as the platform matures.

## Data Download

Users can download raw images, processed images (e.g., segmented gray matter images), and the data metrics described earlier. Download functions are slightly different for public and private datasets, as visually represented by the download component (third from the top) in Fig. 1. Public datasets consist of data from users who have chosen to share their data openly and are accessible only to those who have signed a Data Use Agreement (DUA). In contrast, private datasets include all data that a user has uploaded and processed on the platform and are only accessible to that specific user unless explicitly shared. The user interface for downloading data includes customizable filtering options, allowing users to refine their selections based on specific criteria such as age. These filters help reduce download size and focus on relevant data.

In the data download module of our platform, we provide customizable options for users to streamline data selection based on study-specific requirements. Researchers can select participant data using a toggle switch to limit the inclusion of data according to a specific demographic criterion. Further, displaying histograms of age distribution provides an overview of the selected data to guide the selection of the multisite data. These features allow users to tailor their downloads according to their research needs. Figures S1 and S2 in Online Resource 1 present a snapshot of the download data interface.

By default, all primary and secondary neuroimaging data are included in the download files, but users can more specifically select the secondary images to download based on their needs. We refer to primary data when discussing the original raw data provided by contributors, particularly in the context of image processing, where the primary data (T1-weighted images) are processed to generate data that are typically used in statistical analyses (e.g., segmented gray matter images). Specifically, secondary data are generated when T1-weighted images are processed to generate native space gray matter or normalized and modulated gray matter volume, or cortical thickness data, for example. Users are provided tools to select specific images to minimize data handling demands and optimize storage utilization.

### Asynchronous Processing of Data Requests

**Fig. 3:**
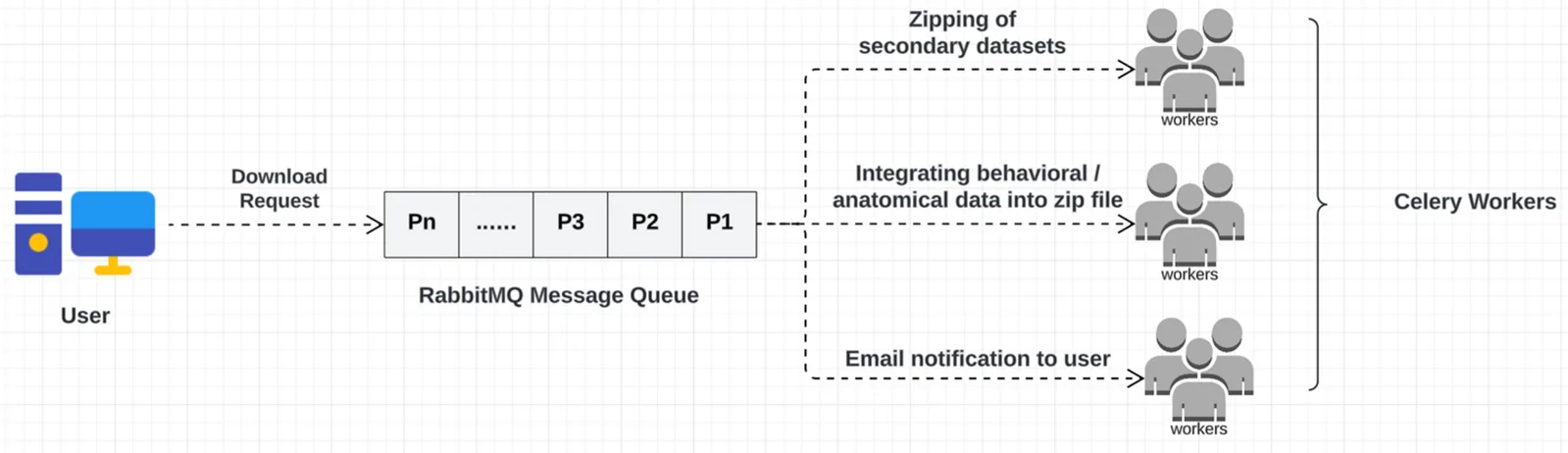
Asynchronous download archive generation process. When users request data, their requests are added to a queue, and Celery workers sequentially handle three tasks: (**1**) Packaging Secondary Datasets, (**2**) Packaging Behavioral/Anatomical Data, and (**3**) Sending the Download link via email

The platform processes download requests asynchronously to handle high user demand for large data volumes (Fig. 3). A backend process manages the download requests through RabbitMQ (RabbitMQ, 2024) in conjunction with Celery (The Celery Project, 2024), a distributed task queue. This combination enables efficient handling of large-scale data processing. Once files are requested, RabbitMQ places the processing tasks in a queue. Celery workers or background processes then handle these tasks asynchronously by pulling them from the queue and processing them. The task scheduler processes these requests on a first-come, first-served basis, preparing the download files. Users can interact with other parts of the platform interface while waiting for their requests to complete. They will receive an email with a link to their requested files upon the completion of data preparation. The RabbitMQ and Celery setup can scale with increasing data demands. As the number of download requests increases, additional Celery workers will be added to handle the increasing load, ensuring that the system continues to perform efficiently, even with high volumes of data. RabbitMQ and Celery allow backend processing to occur independently of user actions, meaning that users can continue to work with the platform without waiting for their requests to be processed.

## Privacy Control of Datasets

Deidentification of shared data is crucial for protecting participant privacy and minimizing the risk of re-identification.

Raw MRI scans may include voxels that can be rendered to visualize facial structures, which could risk participant identification and discourage contributors and institutions from sharing data. This issue becomes particularly relevant as more data is shared openly for scientific advancement. Proper deidentification processes ensure that sensitive facial features, are removed before sharing, protecting participants’ privacy and making data-sharing in open-access repositories safer. However, this process can be challenging due to the variability of MRI scans, including differences in size, shape, contrast, and orientation. Researchers who share the data and the administrators of the sharing platform often must visually inspect the images to verify that the shared data are appropriately de-identified. Performing such manual checks is viewed as an extra burden for contributors or administrators of the sharing platform to ensure data privacy. To address the challenge of time-consuming visual inspection of skull-stripped MRI images, we have designed and integrated a deep learning model (Luo et al., 2025) in our platform. This model assesses the probability that skull-stripping has retained identifiable facial features. If the model detects a higher likelihood of failed de-identification, the images are flagged for further review. Platform administrators are also notified of any potential risk for re-identification so that they can communicate with the contributor and/or remove the data. This deep learning model acts as an efficient filter, flagging images that require additional attention and allowing researchers to focus on problematic scans instead of manually checking every image. Our platform further integrates the FSL defacing tool (Smith et al., 2004), a widely used neuroimaging software, to refine improperly defaced skull-stripped images. Multiple refined image versions are rendered using different erosion radius values, ranging from conservative to more aggressive defacing. These corrected images are then presented to researchers via an interactive interface, allowing them to visually assess and select the version that best retains critical anatomical features while meeting privacy requirements.

## Quality Control of Datasets

Data quality assessment is critical for ensuring the reliability and user confidence in a multi-site retrospective data repository. The platform includes the following functions to facilitate data sharing while providing data quality measures for contributors and allowing data recipients to control for or exclude cases with lower-quality data.

### Data Integrity

Image skull-stripping and defacing methods typically rely on operations such as erosion, where an erosion radius parameter determines the extent of tissue removal. However, default defacing parameters developed for adult images do not work for every image and can remove the tissue of interest. Thus, data de-identification methods can introduce data quality problems that affect image processing and the collection of brain structure metrics described earlier. We developed a deep-learning approach to assess whether voxels representing brain tissue have been mistakenly removed (Luo et al., 2025). This model evaluates the likelihood of brain tissue voxels being removed from an image.Based on higher probability values, if the system flags an image for potential loss of brain tissue, users can take several actions: 1) remove the problematic image from the database if there is significant voxel loss; 2) upload a new image; 3) retain the image if only minor loss is detected; or 4) select an alternative skull-stripped version. This iterative approach minimizes data loss and increases data processing consistency across datasets. 

Poor spatial normalization is another common data quality issue that can lead to misalignment across subjects and compromise the accuracy of group-level analyses. Spatial normalization involves aligning individual brain images to a standardized template image to facilitate voxel-wise brain region comparisons across participants. Differences in brain shape or size can make it difficult for automated normalization algorithms to align brains accurately to the standard template, even if these algorithms have substantially improved by diffeomorphic normalization methods (Mangin et al., 2016). Apart from individual variability, atypical morphology, like congenital malformation, can disrupt the normal anatomy and make it hard for the normalization process to align the individual brain to the template correctly. For example, (Eckert et al., 2016a; Eckert et al., 2019) reported unique cases with atypical morphology corresponding to distinct behavioral patterns of reading disability.

In addition to atypical morphology, image acquisition artifacts and motion artifacts can also compromise the quality and precision of image processing approaches, which can be reflected in poor spatial normalization. One approach for identifying cases with poor spatial normalization is to examine their structural similarity to the image template. The platform converts a spatially normalized 3D image and the template image to single vectors of voxel values that can be correlated, which provides a similarity metric. Images with poor spatial normalization and/or gray matter segmentation are identified as having below-average similarity or Pearson correlation compared to the larger sample. This approach helps to identify images that should be excluded, or at least reviewed, before data analysis. This image similarity metric is also used with an Image Quality Rating metric generated using the CAT12 toolbox, which provides a measure of image contrast that can be low when there is any motion artifact and impacts the quality of image segmentation. Thus, the platform includes data quality metrics that can inform image segmentation quality and spatial normalization.

### Duplicate Images

It is not uncommon for images to be mislabeled or for enrollment of the same participant in multiple studies. The image (dis)similarity approach described in the previous section is also used with the original uploaded images to identify duplicate images in the repository. This process involves comparing each newly uploaded image to all other uploaded images in the database. Images with high correlation values may reflect duplicate images. The platform has a data quality review page where cases can be sorted by image quality metrics, including an image similarity metric to identify images that may be problematic. Figure S4 of Online Resource 1 shows the interface for this review process.

## Data Analysis Resources

Our platform integrates JupyterHub resources, allowing researchers to conduct image and statistical analyses without downloading the data to their local storage, thus streamlining the workflow and limiting the computational demands for users. Researchers can perform interactive analyses using Jupyter Notebooks, which allow them to code, visualize, and document their work. The plotting libraries like Matplotlib, Seaborn, and others make it easier to visualize data directly within the notebooks. This approach also allows several researchers to work on the same dataset, providing a shared environment for them to collaborate. Such collaboration promotes consistency in the analysis pipeline, as all team members use the same environment, tools, and data. Users can save their notebooks and share their analyses with others, allowing their work to be reproducible without concerns about local environment differences across team members.

JupyterHub also allows users to leverage scalable computational resources for tasks such as deep learning or large-scale neuroimaging analysis, making it feasible to handle complex datasets efficiently. Users are responsible for validating their analysis code and the results generated through the platform. Statistical results from the repository data may change over time with the submission of new data to the repository. Use of JupyterHub and archived data ensures consistency and interpretation of results. The Jupyter Notebooks are available to all users, thus allowing for code sharing and user evaluation of results generated with the repository data. Users who access those notebooks and use the code in their projects must provide attribution to the original authors. Clear attribution of code sources enhances research transparency, allowing others to assess the reliability and validity of methods and results. By adhering to these principles, users contribute to a more transparent, reproducible, and ethically responsible research environment.

## Data Access and Governance

As noted earlier, a DUA is mandatory to access data in the repository to ensure appropriate use, contributor acknowledgment, and consideration of enhancements to the repository. That is, the DUA process ensures that the datasets are used responsibly. To support responsible data sharing, project personnel oversee data access requests, monitor for potential misuse, and ensure that uploaded files contain no personal identifiers (e.g., HIPAA identifiers). The DUA is also designed to establish expectations for appropriate use of the DDC platform, while ongoing case-by-case review enables responsive oversight. The platform prompts users and their institution representative to sign the DUA during account registration. Users may also sign the DUA later when they request to download the data. There is a vision for facilitating data access to limit the delay in implementing research plans, and the repository can also be used for educational reasons. Looking forward, we have also established an External Advisory Board with expertise spanning basic science, clinical research, and data sharing to address emerging ethical considerations and guide repository development.

## Limitations/Future Directions

Although the current repository infrastructure does not support longitudinal data processing, this is a priority for future development with a vision for tracking delayed and/or atypical trajectories of reading development. Future updates will also facilitate users with greater flexibility in customizing image-processing pipelines, including options to incorporate demographically appropriate priors and to generate study-specific templates for spatial normalization.

To broaden the image processing capabilities of the platform, work is underway to integrate image processing methods for additional neuroimaging modalities, including functional MRI (fMRI) and diffusion MRI (dMRI). Planned integrations include fMRIPrep (Esteban et al., 2018), which will deliver preprocessed fMRI data with motion-corrected time series, and QSIPrep (Cieslak et al., 2021) to provide white matter microstructure metrics. We are integrating image-processing pipelines that measure similar constructs (e.g., cortical thickness from FreeSurfer and CAT12) so that users can evaluate whether results depend on the processing method. Data repositories benefit from formal ontological organization. We are also integrating ontology-based harmonization methods to strengthen standardization and cross-study comparability. This also facilitates communication with users about what each data point means, how it was collected, and how to best interpret results.

## Conclusion

The DDC platform provides a centralized repository for researchers to access and share data, facilitating collaboration, education, replication, and discovery. The platform addresses challenges such as data validation, annotation, and standardization to optimize data integrity while organizing and combining behavioral, demographic, and neuroimaging datasets. The addition of the JupyterHub resource to the platform allows users to access and analyze data interactively, utilizing various computational tools and libraries. Researchers can access curated datasets, apply advanced analysis techniques, and share their results with the broader community. The ultimate goal is to advance understanding of the neurobiology of reading development and reading disability.

## Supplementary Information

Below is the link to the electronic supplementary material.

Supplementary Material 1 (PDF 704 KB)

## Data Availability

No datasets were generated or analysed during the current study.
