# Supplementary material for: Dyslexia Data Consortium: A Comprehensive Platform for Neuroimaging Data Sharing, Analysis, and Advanced Research in Dyslexia
Source: Neuroinformatics. 2025 Oct 8;23(4):49. doi: 10.1007/s12021-025-09747-0 (PMC12508011; doi:10.1007/s12021-025-09747-0)
Supplement: Supplementary file 1 — Supplementary Material 1 (PDF 704 KB) [file 12021_2025_9747_MOESM1_ESM.pdf]

Dyslexia Data Consortium: A Comprehensive Platform for Neuroimaging Data Sharing, Analysis, and Advanced Research in Dyslexia

Rishikesh V. Phatangare[1*], Mark A. Eckert[2], Li Luo[1], Kenneth I. Vaden Jr.[3], James Z. Wang[1]

1 School of Computing, Clemson University, Clemson, USA; rphatan@g.clemson.edu; lluo2@clemson.edu; jzwang@clemson.edu

2 Department of Otolaryngology - Head and Neck Surgery, Columbia University, New York, USA; me2935@cumc.columbia.edu

3 Department of Otolaryngology - Head and Neck Surgery, Medical University of South Carolina, Charleston, USA; vaden@musc.edu

4 Dyslexia Data Consortium; dyslexiadata@musc.edu
* Correspondence: rphatan@g.clemson.edu

Journal: Neuroinformatics

Supplementary Material

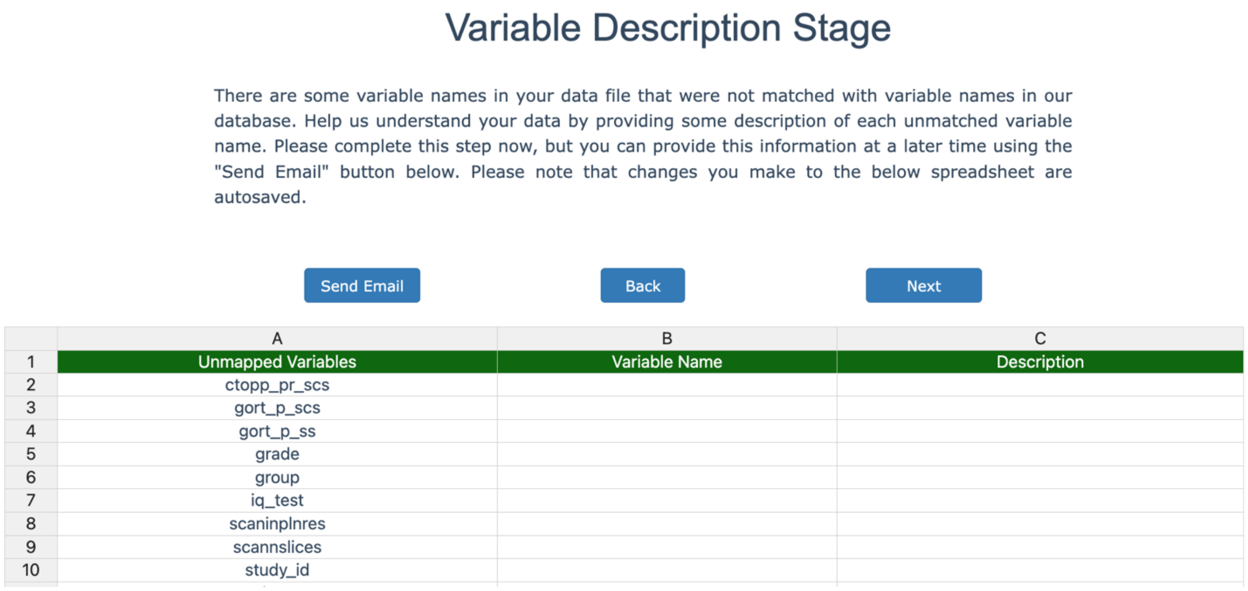

Figure S1 Variable description interface. The data upload stage is designed to match variable names in a contributor's dataset with variable names in the repository database. This interface is used for labeling variables that were not automatically or manually matched during a preceding upload stage. Contributors provide a variable name and a description of the variable.

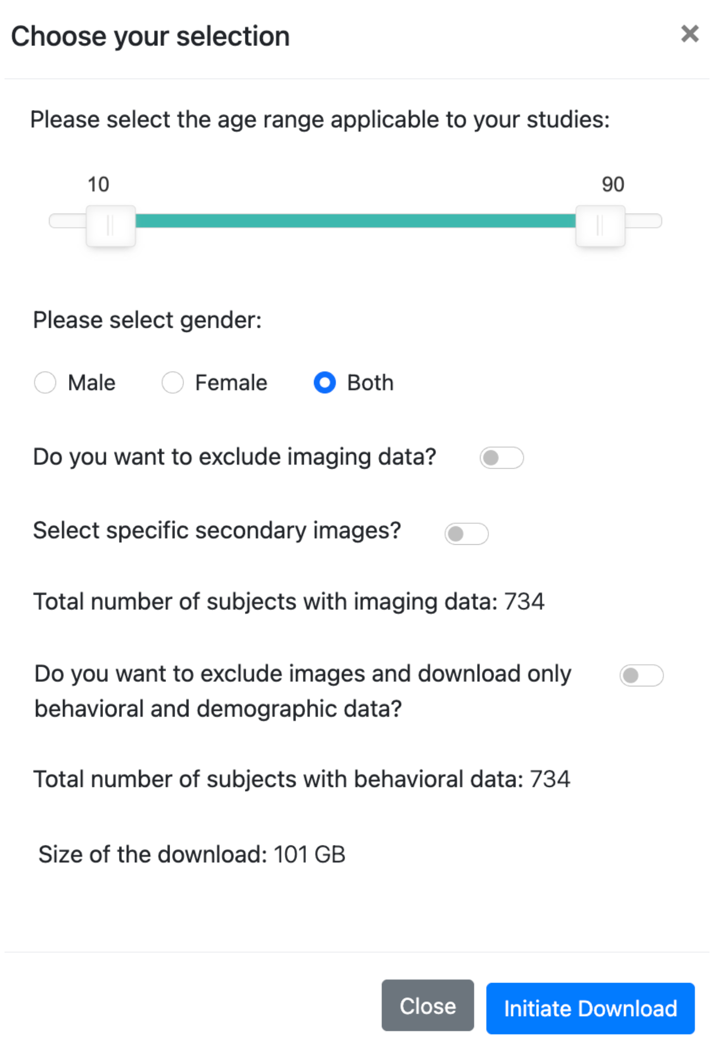

Figure S2 Data Download Interface. Download configuration window showing options to specify study names, age range, and gender. Users can also select a secondary or post-processed images (e.g., segmented gray matter images) and choose to download only the behavioral and demographic data.

Visualization

The histogram visualization below shows a distribution of age categories. You can adjust it by moving the age slider or selecting the radio buttons for male, female, and both categories. By using these controls, you can dynamically modify the chart to focus on specific subsets of the population, providing a clearer understanding of age and gender distribution within your data. To find the exact count of participants under each bin you can always hover your mouse on each of the bars.

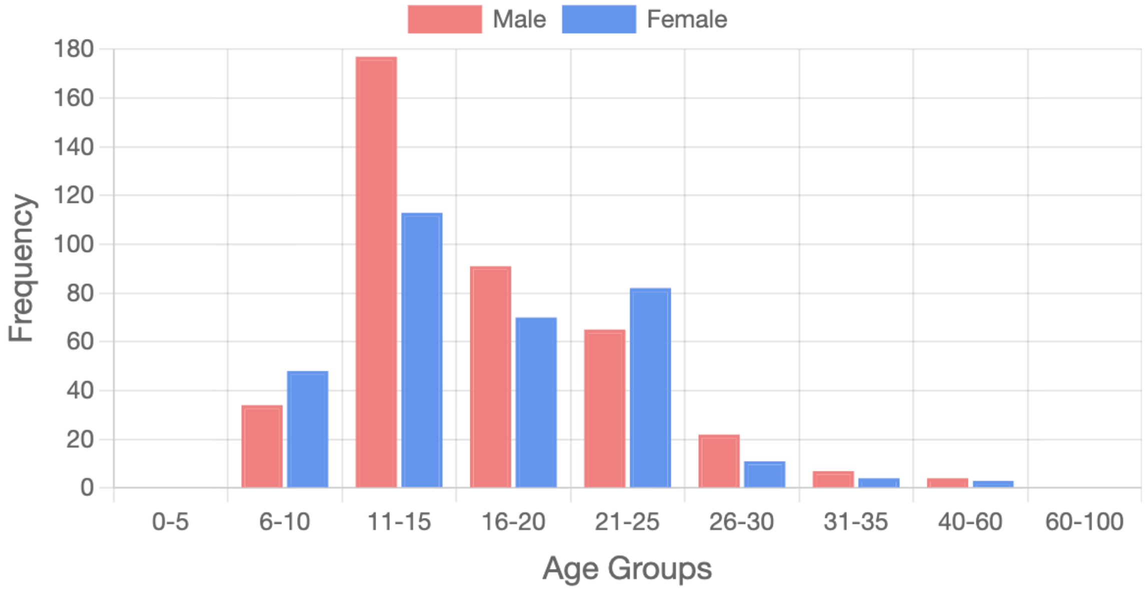

Figure S3 Age and Gender Distribution Visualization. Histogram interface displaying age group frequencies for male (red) and female (blue) participants. Users can adjust the age range using a slider or filter by gender using radio buttons to focus on specific subsets of the population.

Study-name	Subject-ID	Probability brain tissue was cropped	Probability face is present	View	Remove
study1	imgid-4827-sub-1.nii	0.00	0.99	View	Remove
study1	imgid-4828-sub-2.nii	0.00	0.20	View	Remove
study1	imgid-4836-sub-10.nii	0.00	0.16	View	Remove
study1	imgid-4834-sub-8.nii	0.00	0.15	View	Remove
study1	imgid-4839-sub-13.nii	0.00	0.11	View	Remove
study1	imgid-4842-sub-16.nii	0.00	0.09	View	Remove
study1	imgid-4830-sub-4.nii	0.08	0.09	View	Remove
study1	imgid-4846-sub-20.nii	0.01	0.08	View	Remove
study1	imgid-4845-sub-19.nii	0.00	0.08	View	Remove
study1	imgid-4844-sub-18.nii	0.02	0.07	View	Remove

Figure S4 Data Quality Interface. Interface displaying file names with predicted likelihood for the presence of a face in the T1-weighted image and loss of voxels representing brain tissue following defacing. cases. Orange highlighting in the Subject-ID column is used to direct attention to cases where privacy and data quality concerns need to be addressed. The View buttons can be used to visualize those cases and the Remove buttons can be used to remove cases from the repository.

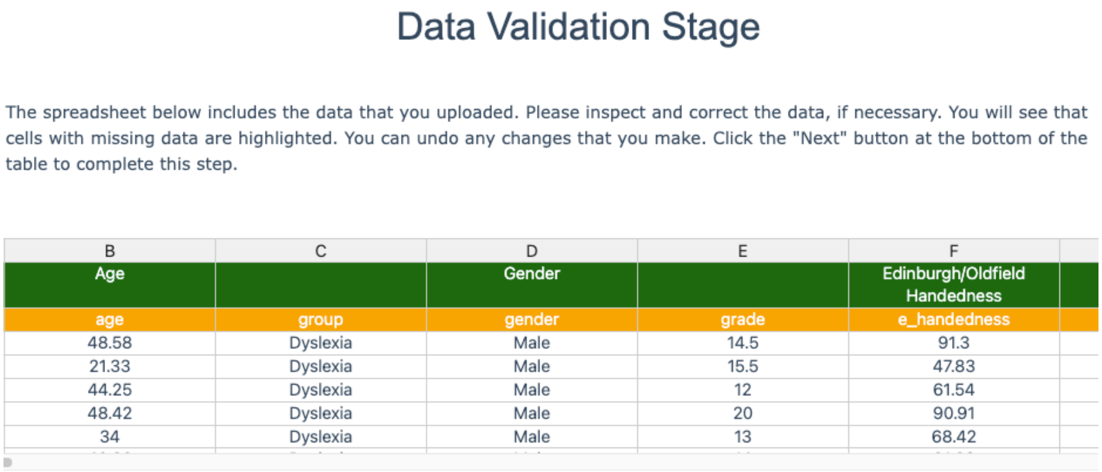

B	C	D	E	F
Age		Gender		Edinburgh/Oldfield Handedness
age	group	gender	grade	e_handedness
48.58	Dyslexia	Male	14.5	91.3
21.33	Dyslexia	Male	15.5	47.83
44.25	Dyslexia	Male	12	61.54
48.42	Dyslexia	Male	20	90.91
34	Dyslexia	Male	13	68.42
[illegible]	[illegible]	[illegible]	[illegible]	[illegible]

Figure S5 Data Validation Step. Data validation interface for inspecting and correcting uploaded dataset entries before submission. The green row represents the database variable names that have been mapped to the contributor's variable names in the orange row. Blank cells indicate that a contributor's variable name was not mapped with a corresponding variable in the database.
